# Changes of Polyphenolic Substances in the Anatomical Parts of Buckwheat (*Fagopyrum esculentum* Moench.) during Its Growth Phases

**DOI:** 10.3390/foods3040558

**Published:** 2014-10-17

**Authors:** Judita Bystricka, Janette Musilova, Jan Tomas, Alena Vollmannova, Jaromir Lachman, Petra Kavalcova

**Affiliations:** 1 Department of Chemistry, Faculty of Biotechnology and Food Sciences, Slovak University of Agriculture in Nitra, Tr. A Hlinku 2, 949 76 Nitra, Slovak Republic; E-Mails: janette.musilova@uniag.sk (J.M.); jan.tomas@uniag.sk (J.T.); alena.vollmannova@uniag.sk (A.V.); petra.kavalcova@gmail.com (P.K.); 2 Department of Chemistry, Faculty of Agrobiology, Food and Natural Resources, Czech University of Life Sciences Prague, Kamýcká 129, 165 21 Prague 6–Suchdol, Czech Republic; E-Mail: lachman@af.czu.cz

**Keywords:** buckwheat, polyphenolics, anatomical parts

## Abstract

In this study the changes of total polyphenolics in different anatomical parts (stems, leaves, flowers and seeds) of common buckwheat (*Fagopyrum esculentum* Moench.) during vegetation period were analysed. The content of total polyphenolics was evaluated in growth phase I (formation of buds), phase II (at the beginning of flowering), phase III (full blossoming) and phase IV (full ripeness). In all growth phases (GP) the stems and leaves were evaluated and statistically significant differences in polyphenolics content between the two parts were confirmed. Statistically significant differences (*p* < 0.01) in polyphenolics content (in GP II and III) between stems and leaves; and between stems and flowers were found. In flowers an average of 13.8 times higher and in leaves 6 times higher concentration of polyphenolics in comparison with stems was measured. In GP III the content of polyphenolics in common buckwheat was following: flowers > leaves > achene > stems. In flowers an average of 11.9 times higher, in leaves 8.3 times higher and in achenes 5.9 times higher contents of polyphenolics compared with stems were found. In GP III and IV (leaves, achenes, stems) the leaves contained in average 20 times higher and achenes 5.6 times higher polyphenolics than stems.

## 1. Introduction

Buckwheat (*Fagopyrum esculentum* Moench.) has been traditionally used as a human food source [1]. In Europe it has been grown since the 16th century. The main producers of buckwheat are China, the Russian Federation, Ukraine, and Kazahstan [2], but it is also produced in Slovenia, Poland and Hungary [3]. The most frequently grown species is common buckwheat (*Fagopyrum esculentum* Moench.) and less common is tartary buckwheat (*Fagopyrum tataricum* L.) [4]. Several hundred molecules with polyphenol structure (*i.e.*, benzene rings with one or more hydroxyl groups) have been identified in edible plants [5]. Polyphenol compounds have been extensively researched in the last decade for health promoting properties, such as their role in the prevention of degenerative diseases, including cancer and cardiovascular diseases [6,7,8,9]. Plant phenolics can be very generally divided into phenolic acids and flavonoids, which are present in the free and conjugated forms [10,11]. The six major flavonoids in buckwheat are rutin, quercetin, orientin, homoorientin, vitexin, and isovitexin [12,13]. Among these compounds, rutin, a flavonol glycoside, has been recognized as a major antioxidant component that accounts for about 85%–90% of the total antioxidant activity [13]. The dominant polyphenol in buckwheat is rutin and buckwheat can be described as an excellent dietary source of rutin [14]. Rutin is a natural flavonoid with antihyperglycemic, antihypertensive and antioxidative properties [15]. Likewise, quercetin (aglycone), a major bioflavonoid of human diet, present in buckwheat, has been identified as a strong antioxidant, anti-angiogenesis, and anticancer [16]. It is also known to reduce the risk of hypertension [17]. Buckwheat is rich in vitamins, especially those of the B group [18], and an important source of macroelements (e.g., K, Na, Ca, Mg) as well as microelements (e.g., Zn, Mn, Cu, Se) [18,19]. In addition, buckwheat seeds are naturally gluten-free and thus, they are currently emerging as healthy alternatives to gluten-containing grains in a gluten-free diet [20]. The total polyphenolics content in buckwheat is discussed in many works. However, the results vary between different researches. This variation could be due to the fact that the buckwheat phenolics can be influenced by geographic origin of seed as well as environmental conditions [21]. To our knowledge there is a lack of data concerning the content of total polyphenols in particular anatomical parts of buckwheat. Based on this, the objective of our study was to determine the total polyphenol content in the anatomical parts (stems, leaves, flowers, seeds) of buckwheat (*Fagopyrum esculentum* Moench.) during all growth phases.

## 2. Experimental Section

### 2.1. Plant Material

Six cultivars of common buckwheat, Pyra, Spacinska, Emka, Kasho, Jana C1 and Hrusowska, were obtained from the Plant Production Research Center, Piestany, Slovak Republic. All six cultivars are registered in the European Union (EU). The investigated buckwheat cultivars were conventionally cultivated in the same locality under the same conditions. The soil type of locality Piestany is degraded chernozem. Chernozem is a black soil, rich in humus (7%–15%) and carbonates. It has a large quantity of nutrients, excellent structure and good water-holding capacity. The annual average temperature of this locality is 9.2 °C, where rejoices in an average annual rainfall of 593 mm, at an altitude of 167 m. Only standard NPK fertilization (N—nitrogen, P—phosphorus, K—potassium), namely granulated mineral fertilizer, N:P:K in relation 4:12:20, amount 50 g/m^2^ was used because of achievement of favourable soil macroelement content. The buckwheat hulled seeds, leaves and stems were manually separated, dried at 105 °C to constant weight (WTC Binder, Tuttlingen, Germany) and powdered (Fritsch Pulverisette, Idar-Oberstein, Germany).

### 2.2. Chemicals and Extraction

The Folin-Ciocalteu reagent and gallic acid were purchased from Merck (Darmstadt, Germany).

Sodium carbonate, methanol and 2,2-diphenyl-1-picrylhydrazyl radical (DPPH) were obtained from Sigma-Aldrich (St. Louis, MO, USA).

Methanol extracts were prepared by adding 100 mL of 80% methanol to 10 g milled sample and was extracted in the Twisselmann apparatus for 12 h. Samples were then filtered with filter paper (130 g/m^2^, Filtrak, Thermalbad Wiesenbad, Germany) and kept at 8 °C for further analysis.

### 2.3. Total Polyphenol Content (TPC)

The total polyphenol content was assessed with the method reported by Lachman *et al.* [22], which employs a reduction reaction of phosphowolframate-phosphomolybdate complex to blue products by phenolic compounds. Briefly, an aliquot of the extract, blank or standard was placed in a 50 mL flask, where the Folin-Ciocalteu assay (2.5 mL) was added and the mixture was allowed to react for 3 min under continuous stirring before a solution of sodium carbonate (7.5 mL) was added and mixed thoroughly. The volume was then made up to 50 mL with distilled water and left standing at room temperature for 2 h. The absorbance was measured at 765 nm using Shimadzu UV-1800 spectrophotometer (Japan). Results were expressed as mg gallic acid equivalents (GAE) per kg fresh weight (FW).

### 2.4. Statistical Analysis

All determinations were done in six replicates. The data was analyzed using the package Statgraphics (multifactorial analysis of variance, LSD-test contrasts, *p* < 0.01).

## 3. Results and Discussion

Buckwheat is a plant that possesses both antioxidant and antidiabetic properties which is attributed to its phenolic contents, like rutin and quercetin [23]. In the present study the dynamics of total polyphenols production in various growth phases and in various anatomical parts of buckwheat (*Fagopyrum esculentum* Moench.) was analysed. Six varieties of buckwheat (Pyra, Spacinska, Emka, Kasho, Jana C1 and Hrusowska) were involved. The analyses were performed during four growth phases (GP I—formation of buds, GP II—the beginning of flowering, GP III—full blossoming, GP IV—full ripeness; GP, growth phases). In all growth phases the stems and leaves were evaluated.

It is well known that polyphenol content and antioxidant property depend on a number of factors such as variety, location and environmental conditions [24,25]. In GP I, of the observed anatomical parts only stems and leaves were available. In our study the highest content of total polyphenols (*p* < 0.01) was in leaves collected in GP IV. Statistically significant differences (*p* < 0.01) in total polyphenol content between observed anatomical parts of buckwheat (stem, leaf) were found (Table 1). Similarly higher polyphenol content in leaves and flowers in comparison with other parts of buckwheat have also been reported by other authors [26,27].

**Table 1:** Changes of total polyphenol content (mg/kg) in stems and leaves of buckwheat during four growth phases.

Anatomical Part	Collection	TP	SD
Stem	GP I	973.38 ^a^	158.64
	GP II	1095.32 ^a^	192.30
	GP III	1653.85 ^a^	292.64
	GP IV	3059.26 ^a^	205.08
Leaf	GP I	6507.92 ^b^	1633.57
	GP II	7322.99 ^b^	1036.83
	GP III	14,188.86 ^c^	4679.57
	GP IV	80,395.53 ^d^	10,231.29

Notes: ^a–d^—different letters in column mean significant difference (*p* < 0.01); TP—total polyphenols; SD—standard deviation; GP—growth phases.

In GP II and III of the observed anatomical parts, the flowers were available. In each variety of buckwheat the highest content of total polyphenols was measured in flowers, followed by leaves and the lowest content was found in stems.

Golisz *et al.* [28] determined higher content of total polyphenols in flowers (9.14 mg/g) and leaves (8.90 mg/g) when compared with stems (2.46 mg/g) of buckwheat. In our study, statistically significant differences (*p* < 0.01) in total polyphenol content between stems and leaves and between stems and flowers were determined. The differences between leaves and flowers remained insignificant (*p* > 0.01, Table 2).

**Table 2:** Changes of total polyphenol content (mg/kg) during GP II and III in stems, leaves and flower of buckwheat.

Anatomical Part	Collection	TP	SD
Stem	GP II	1095.32 ^a^	192.30
	GP III	1653.85 ^a^	292.64
Leaf	GP II	7322.99 ^b^	1036.83
	GP III	14,188.86 ^c^	4679.57
Flower	GP II	15,114.77 ^c^	2389.03
	GP III	19,728.58 ^d^	4477.10

Notes: ^a–d^—different letters in column mean significant difference (*p* < 0.01); TP—total polyphenols; SD—standard deviation; GP—growth phase.

**Figure 1:**
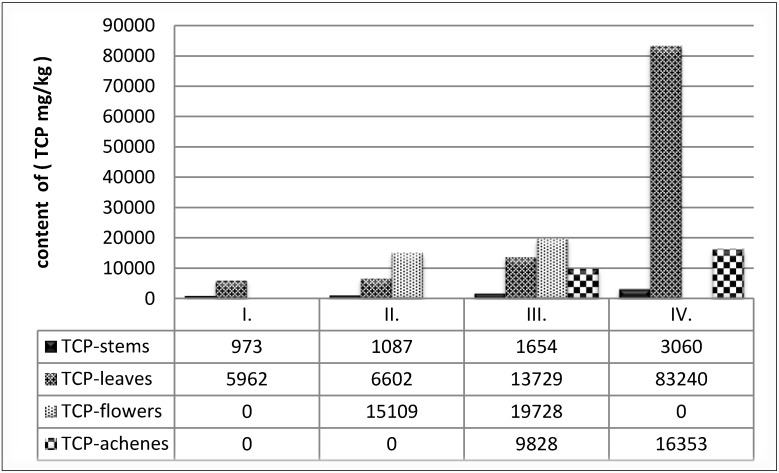
Content of TPC (mg/kg) in all anatomical parts of buckwheat.

All anatomical parts in GP III were analysed. In this phase the content of polyphenolics in common buckwheat was following the order: flowers > leaves > achenes > stems. In flowers an average of 11.9 times higher, in leaves 8.3 times higher and in achenes 5.9 times higher contents of polyphenolics in comparison with stems were found (Figure 1).

These results are in good accordance with some previously reported data. Similar results were published by Lee *et al.* [15]. Authors found the highest content of total polyphenols in flowers of buckwheat. Similar order (flowers > leaves > stems) was also found by Zielińska *et al.* [29] when the content of total flavonoids was determined in different anatomical parts of common and tartary buckwheat plants.

The most important part of buckwheat are achenes. In the full blossoming (third phase of growth) and full ripeness (fourth phase of growth) growth phases, stems, leaves and achenes were evaluated. According to the analysed results, the total polyphenol content in various anatomical parts of buckwheat was following the order: leaves > achenes > stems. In GP III and IV the leaves contained in average 20 times higher and achenes 5.6 times higher polyphenolics than stems.

Obtained results correspond with the findings of Štočková *et al.* [30]. These authors indicated the same order of particular anatomical parts of buckwheat according to decreasing content of polyphenol compounds. The production of phenolic compounds is affected by various factors, such as variety or production conditions [18]. Among all parameters, stress during vegetation period can most greatly affect the production of phenolic compounds [31]. In compliance with Kraus *et al.* [32], the concentration of phenolic compounds is affected by phenological growth phase. Statistical evaluations of total polyphenol content among various anatomical parts of buckwheat are shown in Table 3.

**Table 3:** Changes of total polyphenol content (mg/kg) during GP III and IV in stems, leaves and flower of buckwheat.

Anatomical Part	Collection	TP	SD
Stem	GP III	1653.85 ^a^	292.64
	GP IV	3059.26 ^a^	205.08
Leaf	GP III	14,188.86 ^c^	4679.57
	GP IV	80,395.53 ^e^	10,231.29
Achene	GP III	9828.40 ^b^	2832.69
	GP IV	16,352.37 ^d^	5337.81

Notes: ^a–d^—different letters in column mean significant difference (*p* < 0.01); TP—total polyphenols; SD—standard deviation; GP—growth phase.

Statistically significant difference in total polyphenol content was determined between all observed anatomical parts of buckwheat (stem, leaf, achene). Only a few authors have studied the dynamics of polyphenolic compounds production during the vegetation period. In our experiments, the maximal increase of total polyphenol content in stems and leaves of each variety in GP IV was confirmed. The flowers of buckwheat were evaluated in the beginning of flowering (second phase of growth) and in the full blossoming (third phase of growth). In various varieties of buckwheat the content of total polyphenols in flowers in GP III ranged from 2.36% to 65.7%. In our study, the cultivar dependence of total polyphenol content in individual GP was also evaluated.

The average values of total phenolic contents in stems and leaves in individual buckwheat varieties determined in observed growth stages are shown in Table 4. The significantly (*p* < 0.01) highest content of phenolic compounds was observed during GP IV among all varieties. Variety dependence in total polyphenol content was not statistically confirmed.

**Table 4:** Changes of total polyphenol content (mg/kg) in cultivars of buckwheat (stems, leaves).

Cultivar	Collection	TP	SD
Pyra	GP I	5271.71 ^a,b,c^	4256.51
	GP II	4519.78 ^a,b,c^	3518.08
	GP III	12,395.28 ^d^	10,947.11
	GP IV	46,694.93 ^g^	45,716.86
Spacinska	GP I	3130.30 ^a,b^	2489.37
	GP II	4820.49 ^a,b,c^	3728.07
	GP III	7682.23 ^b,c^	6615.12
	GP IV	35,940.45 ^e^	34,338.14
Kasho	GP I	3197.19 ^a,b^	2419.83
	GP II	4546.88 ^a,b,c^	4012.64
	GP III	7758.91 ^b,c,d^	6535.28
	GP IV	35,917.48 ^e^	34,362.07
Jana C1	GP I	4421.68 ^a,b,c^	3671.39
	GP II	3612.57 ^a,b,c^	2666.61
	GP III	8300.64 ^c,d^	7355.84
	GP IV	46,241.68 ^g^	45,402.49
Hrusowska	GP I	3440.03 ^a,b^	2452.69
	GP II	4033.45 ^a,b,c^	2985.35
	GP III	6263.28 ^a,b,c^	4715.54
	GP IV	41,075.53 ^f^	40,047.18
Emka	GP I	2983.00 ^a^	2124.35
	GP II	3721.74 ^a,b,c^	2698.97
	GP III	5127.79 ^a,b,c^	3288.69
	GP IV	44,494.31 ^f,g^	43,286.86

Notes: ^a–g^—different letters in column mean significant difference (*p* < 0.01); TP—total polyphenols; SD—standard deviation; GP—growth phase.

Statistically significant differences (*p* < 0.01) were found between contents of total polyphenols in stems, leaves and flowers in all buckwheat varieties during GP II and III. The exception was variety Emka without any significant differences (*p* > 0.01) (Table 5).

**Table 5:** Changes of total polyphenol content (mg/kg) in cultivars of buckwheat (stems, leaves, flowers).

Cultivar	Collection	TP	SD
Pyra	GP II	9029.87 ^c,d^	7162.05
	GP III	16,386.45 ^g^	10,792.31
Spacinska	GP II	8952.00 ^c,d^	6744.13
	GP III	11,732.81 ^e,f^	7960.76
Kasho	GP II	7911.99 ^a,b,c^	5881.83
	GP III	10,168.75 ^d,e^	6337.71
Jana C1	GP II	6244.47 ^a^	4407.80
	GP III	11,893.57 ^e,f^	7922.21
Hrusowska	GP II	7952.49 ^a,b,c^	6210.98
	GP III	12,562.61 ^f^	9953.00
Emka	GP II	6975.33 ^a,b^	5226.54
	GP III	8398.39 ^b,c,d^	5470.34

Notes: ^a–g^—different letters in column mean significant difference (*p* < 0.01); TP—total polyphenols; SD—standard deviation; GP—growth phase.

Statistically significant differences in total polyphenol content during GP III and IV among individual varieties are shown in Table 6.

**Table 6:** Changes of total polyphenol content (mg/kg) in cultivars of buckwheat (stems, leaves, achenes).

Cultivar	Collection	TP	SD
Pyra	GP III	13,423.42 ^f^	8945.69
	GP IV	36,574.39 ^d,e^	39,616.15
Spacinska	GP III	8279.61 ^a^	5397.32
	GP IV	29,526.67 ^b,c^	29,160.47
Kasho	GP III	7656.20 ^a^	5265.82
	GP IV	28,561.40 ^b^	29,645.23
Jana C1	GP III	8498.86 ^a^	5932.76
	GP IV	34,706.60 ^d^	40,196.88
Hrusowska	GP III	7611.84 ^a^	4285.42
	GP IV	31,528.22 ^c^	35,084.87
Emka	GP III	5872.26 ^a^	2870.61
	GP IV	38,717.04 ^e^	35,832.48

Notes: ^a–f^—different letters in column mean significant difference (*p* < 0.01); TP—total polyphenols; SD—standard deviation; GP—growth phase.

Our results confirmed a significant increase in total polyphenol content during the vegetation period in each variety and each anatomical part, and correspond with the results of Kalinova and Dadáková [33] who also found lower content of polyphenols in the first growing phase compared to the end of the vegetation period.

Buckwheat is primarily cultivated for the purpose of obtaining the seeds for human consumption, but some published papers [34,35] reported the use of green parts of buckwheat, because rutin is mainly located in flowers and in green parts of the buckwheat plant. According to Bok *et al.* [36], the water extract of buckwheat leaves is used for preparations with anti-obesity effects. Meng *et al.* [37] declared that water extract of buckwheat flowers has inhibitory effect on cancer cells growth, angiogenesis, and also has immunostimulatory effects. Leaves and flowers are utilized as tee mixtures [38], mainly in Japan. Park *et al.* [39] describes that buckwheat flower tea tastes very good, much like green tee.

Buckheat contains a higher content of total polyphenols, in common with cereal and other common vegetables. Klepacka *et al.* [24] reported the highest level of the total polyphenolics in buckwheat (268.8 mg/100 g) and the lowest in pea (35.2 mg/100 g), while the value for winter barley was 107.5 mg/100 g. Comparing with buckwheat the onion family (*Allium*; shallot, onion and garlic) contain a lower content of the total polyphenols [40].

Buckwheat flour is also rich of total polyphenols. Sedej *et al.* [41] reported a higher level of total polyphenols in buckwheat flours (476.3–618.9 μg/g) than in wheat flours (37.1–137.2 μg/g).

Common buckwheat (*Fagopyrum esculentum* Moench.) can be used as raw material for the production of functional foods (buckwheat flour, noodles).

## 4. Conclusions

Buckwheat is one of the best sources of polyphenol compounds. Buckwheat achenes are consumed as bakery products, pasta, biscuits, soups, *etc*. Our results suggest that flowers and leaves are the richest source of total polyphenols, among the anatomic parts of common buckwheat analyzed here, followed by the seeds and stems. Contents of polyphenol compounds depend on the developing phases of the tested buckwheat. When evaluating the dynamics of total polyphenols formation, the maximal increases in the polyphenolic contents of each variety were observed during GP IV, at the end of vegetation period.
